# Therapeutic efficacy of coriander (*Coriandrum sativum*) enriched diets in *Oreochromis niloticus*: effect on hepatic-renal functions, the antioxidant-immune response and resistance to *Aeromonas veronii*

**DOI:** 10.1007/s10695-023-01220-6

**Published:** 2023-07-13

**Authors:** Ahmed Abdou Said, Rasha M. Reda, Mohamed M. M. Metwally, Heba M. Abd El-Hady

**Affiliations:** 1 Department of Pharmacology, Faculty of Veterinary Medicine, Zagazig University, Zagazig, 44511 Egypt; 2 Department of Aquatic Animal Medicine, Faculty of Veterinary Medicine, Zagazig University, Sharkia, Zagazig, 44511 Egypt; 3 Department of Pathology, Faculty of Veterinary Medicine, Zagazig University, Zagazig, 44511 Egypt

**Keywords:** *Aeromonas veronii*, Antioxidant, Coriander, Immunity, Nile tilapia

## Abstract

In this study, the effects of *Coriandrum sativum* to control *Aeromonas veronii* infection in *Oreochromis niloticus* were determined. *Coriandrum sativum* extract (CE) was tested in vitro against *A. veronii* by the disc diffusion assay. In in vivo, 150 *O. niloticus* (from El-Abbassa, Sharkia, Egypt, weighing 34.95 ± 1.98 g) was distributed in five groups (with three replications) in glass aquariums (80 × 40 × 30 cm). The first group (control) was intraperitoneally injected with 0.2 ml of sterilized tryptic soya broth. Groups 2–5 were intraperitoneally challenged with 0.2 ml of *A. veronii* (4.3 × 10^6^). The five groups were administered a basal diet until clinical signs appeared, and then therapeutic feeding (15 days) was followed: the first (CONT) and second (AV) groups were administered a normal basal diet. The third (AV+CP) and fourth (AV+CE) groups were administered diets supplemented with *C. sativum* powder and extract, respectively, each at 30 mg/kg. The fifth group (AV+OT) was administered a diet supplemented with oxytetracycline at 500 mg/kg diet. The results of the in vitro experiment revealed that CE has a zone of inhibition of 43 mm against *A. veronii*. The in vivo results showed that fish administered a therapeutic diet supplemented with CE showed a significant improvement in hematological, biochemical, and immunological parameters, as well as antioxidant capacity (*P* < 0.05) and the pathological findings of the liver and kidney tissues. The current findings supported that the administration of a CE-enriched diet (30 mg/kg) is an eco-friendly strategy for controlling *A. veronii* in *O. niloticus*.

## Introduction

The gram-negative, rod-shaped facultative bacteria known as aeromonads cause many outbreaks that affect farmed fish and aquaculture industry throughout the world (Dien et al. 2023; Reda et al. 2021). They also cause a disease that affects humans, resulting in sepsis, diarrhea, and wound infections (Pessoa et al. 2022). Motile aeromonads in general and *Aeromonas veronii* in particular are the primary causes of Motile aeromonas septicemia (MAS), which is a widespread disease that leads to severe economic losses in the global aquaculture industry (Francis-Floyd 1991; Saharia et al. 2021). Several studies have also shown that *A. veronii*, alone (Abd El Latif et al. 2019; Raj et al. 2019; Reda et al. 2022) and as a co-infection with other strains of bacteria (Dong et al. 2017; Youssuf et al. 2020) or virus (Amal et al. 2018; Suresh et al. 2023), cause summer mortality syndrome in tilapia farms.

To control economic losses due to fish infections, most fish farmers overuse antibiotics without professional management; they often administer antibiotics not approved for fish without a prescription (Preena et al. 2020). In aquaculture, antibiotics are typically administered orally, by immersion, or by adding them to the water. The uncontrolled use of antibiotics and the presence of non-ingested food items and fish excrement can cause antibiotics to persist in the aquatic environment (Serwecińska 2020). The overuse of antibiotics is associated with high risk to public health, including (i) the spread of antibiotic-resistant bacteria and genes; (ii) antibiotic residues in food chains, aquatic products, and ecosystems; and (iii) the elimination of the normal microbiota, which can promote the growth of opportunistic bacteria due to the absence of competition (Romero et al. 2012).

Most studies have investigated sustainable and environmentally friendly approaches to control aquaculture diseases, which can benefit public health by boosting immunity and overall health (Dien et al. 2023; Reverter et al. 2014). Plant-based approaches, often known as phytomedicines, are eco-friendly and safe for aquatic organisms (Tabuti et al. 2003). Most medicinal plants, whether fresh or dried, contain natural bioactive compounds that can be extracted from roots, leaves, bark, flowers, fruits, and seeds (Kuebutornye and Abarike 2020). Many infectious diseases in aquaculture are treated and prevented using medicinal herbs (Dien et al. 2023). Bioactive compounds, such as flavonoids, phenolics, proteoglycans, and polysaccharides, present in medicinal plants prevent aquatic diseases either by enhancing immune responses or by acting as antibacterial agents (Citarasu 2010).

Coriander (*Coriandrum sativum* L.) belongs to the Apiaceae family and is rich in bioactive substances with antioxidant, anti-inflammatory, and antibacterial properties (Mandal and Mandal 2015). Coriander seeds are easily available, have a low cost of production, and contain vital fatty acids such as linoleic acid, minerals, vitamin C, and linalool, which is a potent cellular antioxidant, antibacterial, and anti-inflammatory compound (Farsani et al. 2019). Several studies have investigated the inhibitory effects of *C. sativum* extract (CE) against various pathogenic strains in aquaculture such as *Yersinia ruckeri* in *Oncorhynchus mykiss* (Farsani et al. 2019) and *Aeromonas hydrophila* in *Oreochromis niloticus* and *Catla catla* (Das et al. 2023; Innocent et al. 2011).

However, information on the therapeutic benefits of dietary coriander seed extract in *O. niloticus* is lacking. Therefore, this study evaluated the therapeutic effect of a diet rich in coriander seed powder (CP) or extract (CE) against *A. veronii* in *O. niloticus*. Here, the antibacterial activity of an ethanolic extract of coriander seed against *A. veronii* in vitro was assessed. This study also determined the in vivo efficacy of CP and CE in restoring the altered hematological, biochemical, and immunological profiles, antioxidant capacity, and histopathological findings of *O. niloticus* that were experimentally infected with *A. veronii*.

## Materials and methods

### *Aeromonas veronii* strain

*Aeromonas veronii* (131TF-ID) used in this study was isolated from the naturally infected fries of tilapia in Idku, Beheira Governorate, Egypt. The isolate was identified as *A. veronii* via rRNA gene sequence and was recorded in the GenBank database (accession number: MN967136) (Reda et al. 2022). The LD_50_ of *A. veronii* was estimated in our laboratory and found to be 4.3 × 10^6^ CFU/ml (Reda et al. 2022). *Aeromonas veronii* was grown in tryptic soya broth (TSB) and agar (TSA) at 30 °C. The bacterial stock was preserved in TSB containing 15% glycerol at −80 °C until needed.

### Coriander (*Coriandrum sativum*) collection and preparation

The coriander seeds used in this study were purchased from a traditional market in Zagazig, Egypt. The seeds were thoroughly washed with water before drying for 15 days at 30 ± 2 °C away from direct sunlight. The dried seeds were milled with a pestle and mortar to a very fine powder. One part of the milled powder sample was stored in a sealed vial at 4 °C and used as a powder (CP). The other portion of the milled powder sample was used for extraction following a method described by Ahmed et al. (2020) to prepare ethanolic coriander extract (CE).

### Determination of the antibacterial activity of *Coriandrum sativum* extract against *A. veronii*

The antibacterial activity of CE against *A. veronii* was determined using the disc diffusion method (Tenover 2019). For preparing the *C. sativum* extract discs (CE discs), Whatman No. 1 filter paper was used to prepare discs (5-mm diameter in diameter), which were sterilized at 121 °C for 15 min. Then, the discs were immersed in CE for 1 h and dried in the oven at 50 °C for 1 h. On the surface of TSA, 100 μl of the *A. veronii* cell solution (10^7^/ml using McFarland standard tubes) was inoculated and evenly spread using a sterile glass spreader. Then, the CE discs were placed on the surface of the agar plates and incubated at 30 °C for 24 h. Discs soaked in distilled water served as the negative control, while oxytetracycline discs (30 mg; HiMedia) served as the positive control. This test was conducted in triplicate. To determine the size of the inhibition zones beyond the paper disc, the diameters of the growth-free zones around the discs were measured, and the diameter of the paper disc was subtracted from the diameter of the inhibition zone. The activity was considered to be strong when inhibition zone was more than 15 mm, moderate when the inhibition zone was 10–15 mm, and weak when the inhibition zone was less than 10 mm (Pachanawan et al. 2008).

### Determination of bioactive compounds using gas chromatography-mass spectrometry (GC-MS)

The bioactive compounds in the CE were identified via GC-MS (Agilent Technologies); a gas chromatograph (7890B) and a mass spectrometer detector (5977A) were used at the Central Laboratories Network, National Research Centre, Cairo, Egypt. The compounds were identified by comparing the spectrum fragmentation pattern with those stored in Wiley and the NIST Mass Spectral Library (Dakhlaoui et al. 2022; Oprean et al. 2001).

### Preparation and experimental protocol of the medicated diet

Four experimental diets were administered that met the nutritional needs of tilapia fish, as recommended by the National Research Council (NRC 2011) (Table 1). The first diet (CONT) served as the control basal diet without any supplementation. The second and third diets were supplemented with CP and CE, respectively, each at 30 mg/kg. The CP and CE levels administered were established based on the findings of study on Nile tilapia by Ahmed et al. (2020). The fourth (OT) diet was the antibiotic diet, which was supplemented with 500 mg/kg oxytetracycline (Pharma Sweed, Egypt) (Hashem et al. 2022). Each diet component was mixed, ground, and pelletized to a diameter of 1.5 mm and dried for a day at room temperature. The pellets were stored at 4 °C until use.

**Table 1:** Composition of experimental diets (g/kg)

	Diets			
	Control diet*(CONT)	Diet 2(CP)	Diet 3(CE)	Diet 4(OT)
Diet ingredients (%)				
Fish meal (65.4% CP)	400	400	400	400
Soybean meal (44%)	200	200	200	200
Yellow corn	130	130	130	130
Wheat flour	150	150	150	150
Wheat bran	20	20	20	20
Fish oil	70	70	70	70
Monocalcium phosphate	20	20	20	20
^(1)^Vitamin mixture	4.5	4.5	4.5	4.5
^(2)^Mineral mixture	5.5	5.5	5.5	5.5
*Coriandrum sativum* seed powder	-	0.03	-	-
*Coriandrum sativum* seed extract	-	-	0.03	-
Oxytetracycline	-	-	-	0.5
Calculated composition (% DM)				
Crud protein	38.90	38.90	38.90	38.90
Crude fat	10.50	10.50	10.50	10.50
Ash	5.84	5.84	5.84	5.84

*Control normal diet (NRC 2011)

^(1)^Vitamin mix (IU or mg kg diet): vitamin A, 16000 IU; vitamin D, 8000 IU; vitamin K, 14.72; thiamin, 17.8; riboflavin, 48; pyridoxine, 29.52; cynocobalamine, 0.24, tocopherols acetate, 160; ascorbic acid (35%), 800; niacinamide, 79.2; calcium-D- pantothenate,73.6; folic acid, 6.4; biotin, 0.64 L-carnitine, 100

^(2)^Mineral mix (mg kg diet): Cu (CuSO4), 2.0; Zn (ZnSO4), 34.4; Mn (MnSO4), 6.2; Fe (FeSO4), 21.1; I (Ca (IO3)2), 1.63; Se (Na2SeO3), 0.18; Co (CoCl2), 0.24; Mg (MgSO4.H2O), 52.7

One hundred and fifty *O. niloticus* (mean ± SE; 34.95 ± 1.98 g), all of which appeared to be in good health, were obtained from a private fish farm in El-Abbassa, Sharkia, Egypt. The fish were placed in glass aquariums (80 × 40 × 30 cm) with 60 L of dechlorinated tap water to acclimate for 15 days. After acclimatization, the fish were divided into five groups of three, each with 10 fish (10 fish/replicate, 30 fish/group). The first group (CONT) was injected with 0.2 ml of sterilized tryptic soya broth. Similarly,0.2 ml of *A. veronii* (4.3 × 10^6^) was intraperitoneally injected into the fish in the other four experimental groups (Reda et al. 2022). The five groups were administered a basal diet until clinical symptoms manifested (such as hemorrhagic spots, loss of scales, and fin rot). After clinical signs appeared, the fish were fed a medicated diet as follows: the first group (CONT) and the second group (AV) were administered a normal basal diet without any supplementation. The third (AV+CP), fourth (AV+CE), and fifth (AV+OT) groups were administered CP, CE, and OT diets, respectively. Based on the number of surviving fish, the amount of food provided in each aquarium was calculated as 3% of the total live fish weight/aquarium and provided to the fish three times daily (800, 1200, and 1600 h). All fish were fed by hand very slowly to ensure ingestion of the introduced pellets until no fish accepted any more pellets (Wang et al. 2006). The amount of actual food consumed daily/aquarium was calculated by subtracting the calculated amount of food/aquarium from the actual food that was eaten/aquarium.

During the experiment, the water parameters were monitored and kept within recommended values following the method described by Boyd and Tucker (2014). The dissolved oxygen (electronic oxygen-meter, Oxi-330, Entech Co., Thailand) was adjusted to 6 ± 0.5 mg/L; the water temperature (digital thermometer WT-2, T-Bota Scietech Instruments & Equipment Co., China) was 27.5 ± 1 °C; and the concentration of unionized ammonia (DREL/2 HACH kits, HACH Co., Loveland, CO, USA) was 0.23 ± 0.05 mg/L.

The clinical signs and postmortem findings were recorded daily. The behavior of the fish was monitored during the 15-day trial between 800 and 1600 h each day using a stopwatch and a video camera to record and score its frequency (Altmann 1974). Gross measurements of the changes in swimming activity, such as whether quick or sluggish, were recorded by measuring the mean swimming frequency and the mean time (s) for 8 h daily. Additionally, if there were any changes in the position of the fish in the water column as surfacing swimming or resting on the bottom were recorded (Chen et al. 2001; Little and Finger 1990). The startle reflex was also examined, including fish reflexes to unexpected tapping on one side of the aquarium and attempts to catch them with a net (Fetcho 2009; Neo et al. 2015). Fish feeding behavior and feed consumption are used to assess appetite (Wei et al. 2021; White et al. 2016). The actual feed consumption was calculated, and the feeding behavior was scored following the method described by Wei et al. (2021) as follows: (−) indicated that the fish consumed all available pellets and moved freely around the pellets; (+) indicated that most fish moved toward the pellets and then returned to their position; (++) indicated that most fish only consumed pellets that fell in front of them; and (+++) indicated that most fish did not respond to food.

### Blood sample collection and hematological analysis

To collect samples, the fish were sedated using 100 mg/L benzocaine solution (Al-Nasr Pharmaceutical Chemicals, Co.). The complete blood count was examined in nine samples per group, which were collected using 1-mlethylenediaminetetraacetic acid-rinsed syringes and a Sysmex XT-2000iV automated hematology analyzer. For analyzing the biochemical and immunological parameters, nine blood samples were collected from the caudal peduncle without using any anticoagulant for serum separation by centrifugation at 3000× g.

### Biochemical analysis

Following the method described by Reitman and Frankel (1957), the activity of the serum enzymes alanine aminotransferase (ALT), aspartate transaminase (AST), and alkaline phosphatase (ALP) was measured. Urea and creatinine levels were determined by the Colorimetric method described by Patton and Crouch (1977) and Owen et al. (1954), respectively.

### Antioxidant activity and non-specific immune parameters

Using commercial enzyme-linked immunosorbent assay (ELISA) test kits (Cusabio Biotech Co., Ltd.) and following the manufacturer’s recommendations to measure the blood levels of total antioxidant capacity (TAC), superoxide dismutase (SOD), and catalase (CAT), spectrophotometric analysis was performed to measure the concentration of nitric oxide (NO (Rajaraman et al. 1998). The serum lysozyme activity (LYZ) was measured using a turbidimetric technique described by Ellis (1990).

### Histopathological examination

Regarding the histopathological evaluation, the fish were humanly euthanized by an overdose of benzocaine solution. The representative hepatic and renal tissue specimens from 10 randomly selected tilapias per group were sampled, rinsed in normal saline, and fixed in 10% neutral-buffered formalin solution for 24 h. After fixation, the specimens were thoroughly washed in distilled water, dehydrated by passing through ascending series of ethyl alcohol (70–100%), cleared in Histo-Clear II (Scientific Laboratory Supplies Ltd., UK), impregnated and embedded in paraffin wax, sectioned into slices (4 μm thick), and stained with hematoxylin and eosin (Suvarna et al. 2018). The slides were examined under a light microscope, and five randomly selected non-overlapping fields (10×) per fish were captured using a digital camera. Next, these images were analyzed to determine the frequency of lesions (FQ), establish a quantitative multiparametric lesion scoring method, and calculate the liver and kidney indices (the higher the index the worse the pathological condition of the organ) among the groups following the method suggested by Bernet et al. (1999). The recoded hepatic and renal histopathological alterations (alt) were classified into five reaction patterns (rp), including inflammatory, circulatory, regressive, progressive, and neoplastic. Next, every alteration within each pattern was assigned a score value (*a*) and an importance factor (*w*), where the score value denoted the degree of the alteration and its value ranged between zero (absence of the lesion) and six (diffuse lesion), whereas the importance factor denoted the seriousness of the alteration and its value ranged between one (minor importance) and three (major importance). Finally, the frequency of lesions and liver and kidney indices were calculated by the following formulae:

FQ (%) = N_lesion_ × N_total_^−1^ × 100.

here, Nlesion, fish exhibited a lesion; N_total_, the total number of fish in the group.

Organ index (I_organ_) = Σrp Σalt (aorg rp alt × worg rp alt).

### Statistical analysis

The Shapiro-Wilks test was conducted to determine the normality and homogeneity of all data before statistical analysis was performed. Using IBM® SPSS® Statistics version 25, the data were evaluated via ANOVA followed by Duncan’s post hoc analysis to determine significant differences between the experimental groups. The results are presented as the mean ± standard error (SE). All differences between groups were considered to be statistically at *P* < 0.05.

## Results

### *Coriandrum sativum* seed extract GC-MS analysis

In Table 2, the principal bioactive compounds identified by GC-MS analysis in the CE are listed together with their retention times (RT) and area percentages. Many bioactive compounds have been found in the CE, according to the chromatograms GC-MS results. The results indicated that L-linalool was the main bioactive compound with the highest peak area % (92.52) at 10.107 retention time (RT, min).

**Table 2:** GC-MS chromatogram *Coriandrum sativum* seed retention time (min) and area (%) of the various compounds assigned in the extract

Retention time (RT, min)	Compound name	Molecular formula	Peak area %
9.1	cis-linalol oxide	C_10_H_18_O_2_	1.73
9.54	trans-linalool oxide	C_10_H_18_O_2_	1.7
10.107	L-linalool	C_10_H_18_O	92.52
10.982	Bicyclo[2.2.1]heptan-2-one, 1,7,7-trimethyl-, (1S)-	C_10_H_16_O	1.5
11.623	1-BORNEOL	C_10_H_18_O	0.43
12.064	Silabenzene	C_5_H_6_Si	0.19
12.579	3-methylene-7,11,15-trimethylhexadecadiene-2-hydroperoxide	C_20_H_36_O_2_	0.12
14.782	Dodecane, 2,6,11-trimethyl-	C_15_H_32_	0.1
20.212	Decane, 2,9-dimethyl-	C_12_H_26_	0.1
29.367	Pentadecane, 7-(bromomethyl)-	C_16_H_33_Br	0.13
34.746	Pentadec-7-ene, 7-bromomethyl-	C_16_H_31_Br	0.11
37.973	Phenol, 2,2′-methylenebis[6-(1,1-dimethylethyl)-4-methyl-	C_23_H_32_O_2_	1.35

### Antibacterial activity of *C. sativum* extract

**Fig. 1:**
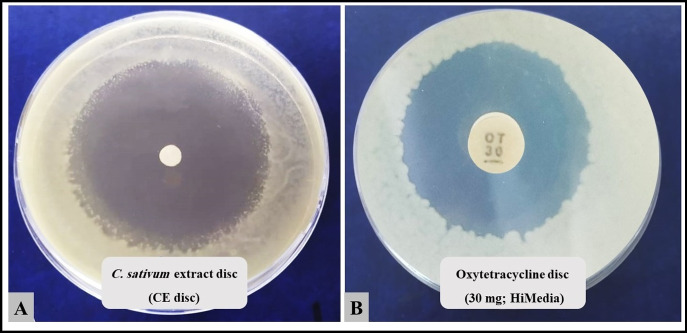
The antimicrobial activity of *Coriandrum sativum* extract against *Aeromonas veronii*

By quantifying the diameter of growth inhibition zones (mm), the antibacterial activity of CE was assessed using the disc diffusion method. CE was proven to be efficient against the fish disease *A. veronii*. The extract of *C. sativum* has a 43-mm zone of inhibition. Oxytetracycline, an antibiotic used in sensitivity testing, produced a zone of inhibition measurement of 48.3 mm (Fig. 1).

### Clinical signs, behavioral observations, and postmortem findings

**Fig. 2:**
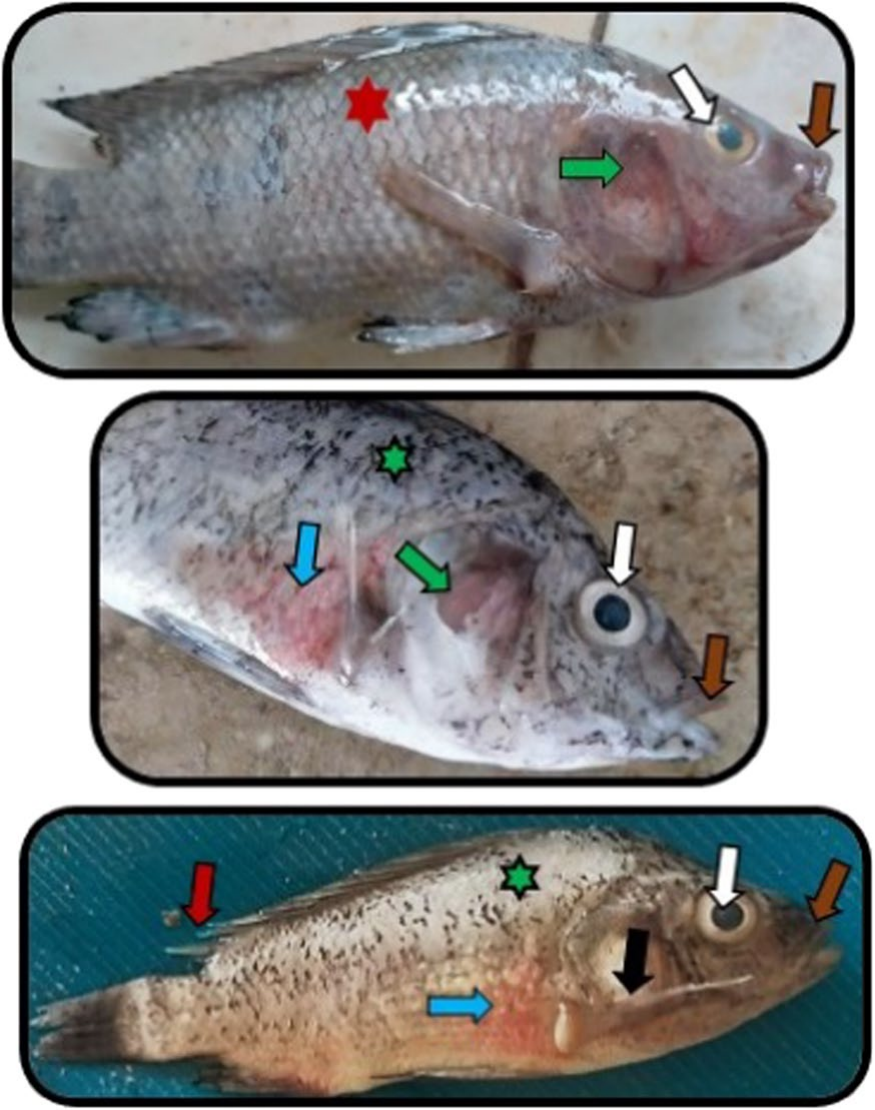
Main symptom of fish from AV group (infected fish fed with normal diet) showing high mucus secretion (red star), eye turbidity (white arrow), erosion of the operculum (green arrow), loss of the scales (green star), hemorrhagic spots (blue arrow), erection in the pectoral fin (black arrow), open mouth (brown arrow), and fin rot (red arrow)

Comparatively to other treatment groups, the AV group displayed observable clinical symptoms and distress manifestations (Fig. 2 and Table 3). Fish showed high mucus secretion, eye turbidity, erosion of the operculum, loss of the scales, hemorrhagic spots, erection in the pectoral fin, and fin rot. These fish also do not respond to knocking on one side of the aquarium, swim in surface water, and are easily caught. Additionally, the amount (g) of actual feed consumed by the AV group was significantly lower (4.02 ± 0.31) than it was for the CONT group (10.15 ± 0.25), and most fish do not respond to food (52.63%). The significant postmortem findings of the AV group included pale gills and an enlarged and congested liver, spleen, and kidney. In the treated groups, the symptoms and postmortem findings listed above were noticeably diminished in AV+CE followed by AV+CP and AV+OT. AV+CE group showed a significant increase in the amount (g) of actual feed consumed (8.45 ± 0.41) followed by AV+CP (6.05 ± 0.48) and AV+OT (6.20 ± 0.28) compared with CONT. The AV group had the highest mortality rate (36.66%), followed by the AV+CP (20%) and AV+OT (16.66%) groups, while the lowest rate (10%) was recorded in the AV+CE group.

**Table 3:** Survival rate and behavior changes of *Aeromonas veronii*-infected *Oreochromis niloticus* treated for 15 days with *Coriandrum sativum* seed powder or its extract

Clinical signs	Experimental groups					AV+OT
		CONT	AV	AV+ CP	AV+CE	
Survival rate	%	100	63.33	80	90	83.33
Rapid and surface swimming	Frequency %	0	57.89	12.50	3.70	16
	Score	-	+++	++	+	++
Low of appetite and feed intake	Frequency %	0	52.63	12.50	7.40	12
	Score	-	+++	++	+	++
Loss of reflexes (knocking on one side of the aquaria and escape when try to catch fish)	Frequency %	0	42.10	8.33	7.40	12
	Score	-	+++	+	+	++
Respiratory manifestation (rapid operculum movement and opening mouth to gasp air)	Frequency %	0	57.89	12.50	7.40	16
	Score	-	+++	++	+	++

CONT (control group), fish not infected and fed with normal diet

AV: infected fish fed with normal diet

AV+CP: infected fish fed with diet supplemented with *Coriandrum sativum* seed powder (CP) at 30 mg/kg

AV+CE: infected fish fed with diet supplemented with *Coriandrum sativum* seed extract (CE) at 30 mg/kg

AV+OT: infected fish fed with diet supplemented with oxytetracycline (OT) at 500 mg/kg diet

The symptoms were observed, and its score was established as follows: (−) no; (+) weak; (++) moderate; (+++) severe

### Hematological and biochemical parameters

The RBCs, Hct, Hgb, total protein, globulin, WBCs, lymphocyte, and neutrophil levels in AV group showed a significant decreasing trend from the control non-infected group (Table 4). In contrast, as compared to the CONT group, the levels of the same pervious mentioned parameters were restored in AV+CE followed by AV+CP and AV+OT but not reaching CONT group levels.

**Table 4:** Erythrogram and leukogram of *Aeromonas veronii*-infected *Oreochromis niloticus* treated for 15 days with *Coriandrum sativum* seed powder or its extract

Parameters	CONT	AV	AV+CP	AV+CE	AV+OT	*P*-value
Erythrogram						
RBCs (10^12^/l)	2.47 ± 0.17^a^	0.61 ± 0.06^c^	1.04 ± 0.03^b^	1.20 ± 0.063^b^	0.92 ± 0.15^bc^	0.000
Hct (%)	23.85 ± 3.60^a^	6.66 ± 0.69^c^	13.26 ± 0.53^b^	17.47 ± 0.83^b^	12.16 ± 1.68^bc^	0.001
Hgb (g/dl)	7.75 ± 0.83^a^	3.05 ± 0.50^c^	5.23 ± 0.49^b^	5.13 ± 0.29^b^	5.16 ± 0.53^b^	0.003
MCV (fl)	96.46 ± 14.29^b^	112.06 ± 18.60^ab^	127.53 ± 1.91^ab^	145.49 ± 0.72^a^	133.07 ± 7.49^ab^	0.074
MCH (pg)	31.88 ± 4.81^c^	49.10 ± 4.60^ab^	50.14 ± 3.23^ab^	42.84 ± 1.96^bc^	57.06 ± 3.81^a^	0.01
MCHC (g/dl)	33.67 ± 5.31	47.41 ± 11.29	39.36 ± 2.77	29.44 ± 1.29	43.12 ± 3.72	0.287
Platelets (×10^3^/mm^3^)	35.00 ± 5.19^a^	10.09 ± 3.46^b^	27.33 ± 10.39^ab^	18.33 ± 1.40^ab^	20.03 ± 3.58^ab^	0.093
Total protein (g/dl)	6.6 ± 0.26^a^	2.50 ± 0.11^d^	4.06 ± 0.08^c^	5.46 ± 0.21^b^	4.13 ± 0.18^c^	0.000
Albumin (g/dl)	3.20 ± 0.15^a^	1.82 ± 0.034^cd^	1.96 ± 0.02^bc^	2.30 ± 0.11^b^	1.47 ± 0.24^d^	0.000
Globulin (g/dl)	3.40 ± 0.37^a^	0.67 ± 0.09^c^	2.10 ± 0.09^b^	2.82 ± 0.37^ab^	2.66 ± 0.20^ab^	0.000
Leukogram						
WBCs (×10^3^/mm^3^)	25.02 ± 0.86^a^	12.99 ± 0.65^d^	18.75 ± 0.17^c^	20.79 ± 0.37^b^	18.42 ± 0.14^c^	0.000
Lymphocyte (×10^3^/mm^3^)	22.58 ± 0.54^a^	11.79 ± 0.54^d^	17 ± 0.18^c^	18.85 ± 0.35^b^	16.66 ± 0.06^c^	0.000
Neutrophil (×10^3^/mm^3^)	1.05 ± 0.02^a^	0.24 ± 0.03^d^	0.48 ± 0.04^bc^	0.6 ± 0.05^b^	0.44 ± 0.04^c^	0.000
Eosinophil ((×10^3^/mm^3^)	0.43 ± 0.08^a^	0.31 ± 0.04^ab^	0.36 ± 0.02^ab^	0.37 ± 0.04^ab^	0.20 ± 0.05^b^	0.115
Basophil (×10^3^/mm^3^)	0.04 ± 0.01	0.02 ± 0.008	0.04 ± 0.005	0.03 ± 0.003	0.01 ± 0.003	0.140
Monocyte (×10^3^/mm^3^)	0.91 ± 0.28	0.61 ± 0.24	0.85 ± 0.03	0.92 ± 0.03	1.09 ± 0.09	0.453

Values with different superscripts within rows are significantly different (*P* < 0.05)

CONT (control group), fish not infected and fed with normal diet

AV: infected fish fed with normal diet

AV+CP: infected fish fed with diet supplemented with *Coriandrum sativum* seed powder (CP) at 30 mg/kg

AV+CE: infected fish fed with diet supplemented with *Coriandrum sativum* seed extract (CE) at 30 mg/kg

AV+OT: infected fish fed with diet supplemented with oxytetracycline (OT) at 500 mg/kg diet

*RBCs* red blood cells, *Hct* the hematocrit, *Hgb* hemoglobin, *MCV* mean corpuscular volume, *MCHC* mean corpuscular hemoglobin concentration, *WBCs* white blood cells

**Fig. 3:**
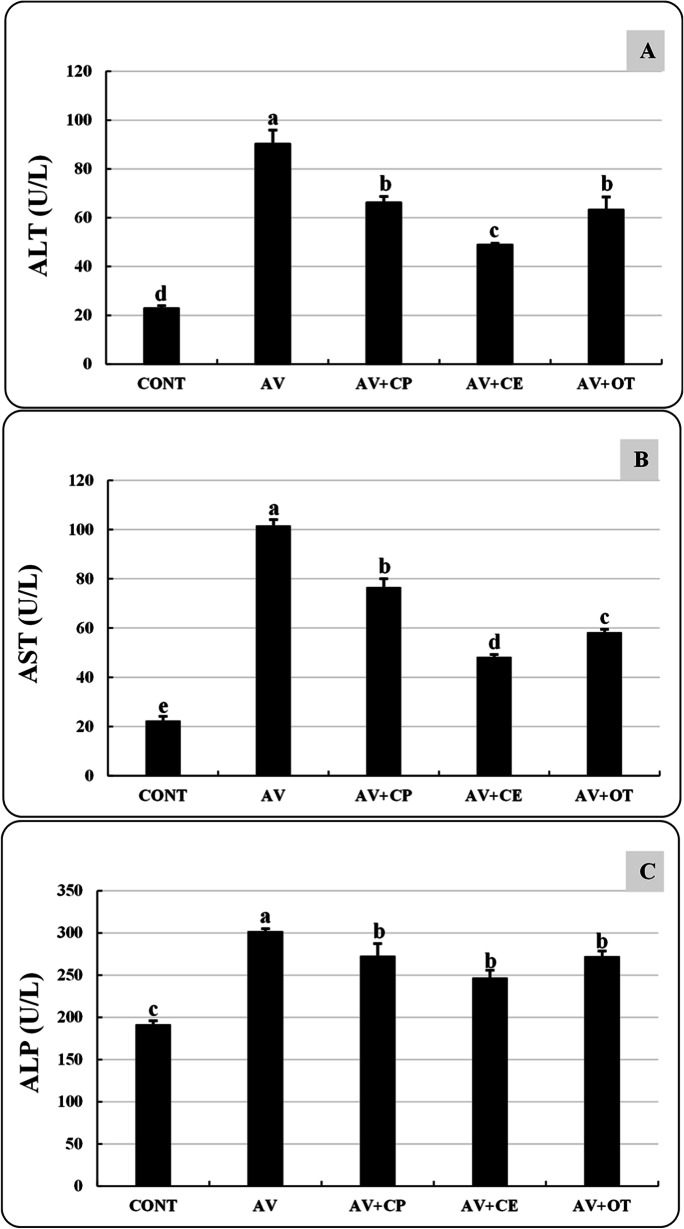
Alanine transaminase (ALT, U/L) (**A**), aspartate transaminase (AST, U/L) (**B**), and alkaline phosphatase (ALP, U/L) (**C**) of *Aeromonas veronii*-infected *Oreochromis niloticus* treated for 15 days with *Coriandrum sativum* seed powder or its extract. The bars with different superscripts (a, b, c, d, and e) are significantly different (*P* < 0.05, one-way ANOVA). CONT (control group) fish not infected and fed with basal diet. AV: infected fish fed with basal diet. AV+CP: infected fish fed with diet supplemented with *Coriandrum sativum* seed powder (CP) at 30 mg/kg. AV+CE: infected fish fed with diet supplemented with *Coriandrum sativum* seed extract (CE) at 30 mg/kg. AV+OT: infected fish fed with diet supplemented with oxytetracycline (OT) at 500 mg/kg diet

**Fig. 4:**
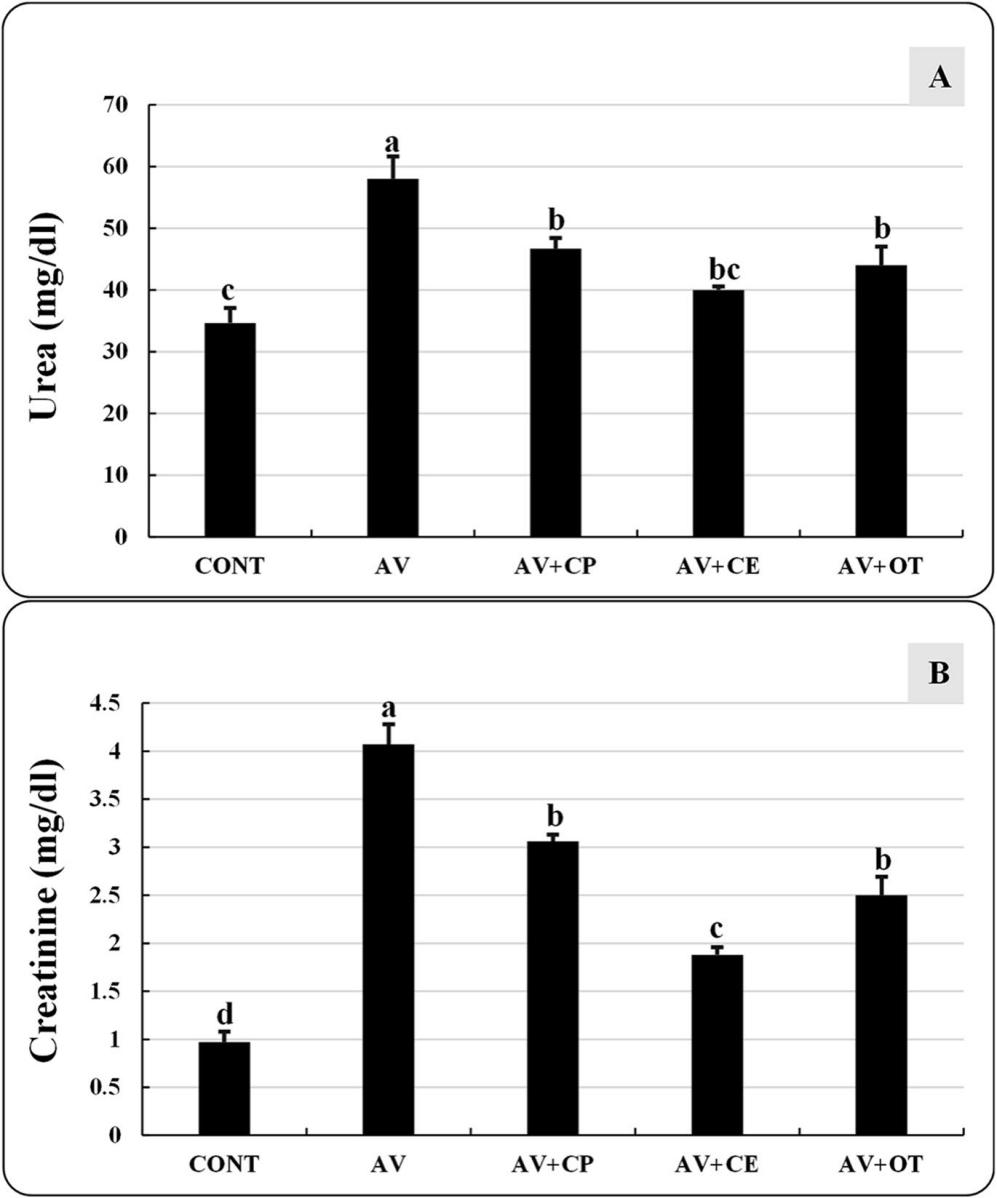
Urea (mg/dl) (**A**) and creatinine (mg/dl) (**B**) of *Aeromonas veronii*-infected *Oreochromis niloticus* treated for 15 days with *Coriandrum sativum* seed powder or its extract. The bars with different superscripts (a, b, c, and d) are significantly different (*P* < 0.05, one-way ANOVA). CONT (control group) fish not infected and fed with basal diet. AV: infected fish fed with basal diet. AV+CP: infected fish fed with diet supplemented with *Coriandrum sativum* seed powder (CP) at 30 mg/kg. AV+CE: infected fish fed with diet supplemented with *Coriandrum sativum* seed extract (CE) at 30 mg/kg. AV+OT: infected fish fed with diet supplemented with oxytetracycline (OT) at 500 mg/kg diet

The *A. veronii*-infected untreated group (AV) has significantly elevated levels of ALT, AST, ALP, urea, and creatinine than the CONT group. Significant improvements were observed in the levels of ALT, AST (Fig. 3), and creatinine (Fig. 4) in AV+CE, but these improvements were not at the same levels as those shown in the CONT group. Although the levels of urea and ALP in AV+CP, AV+CE, and AV+OT have improved noticeably, there are no discernible variations between the levels of these parameters in these groups.

### Oxidant/antioxidant status

**Fig. 5:**
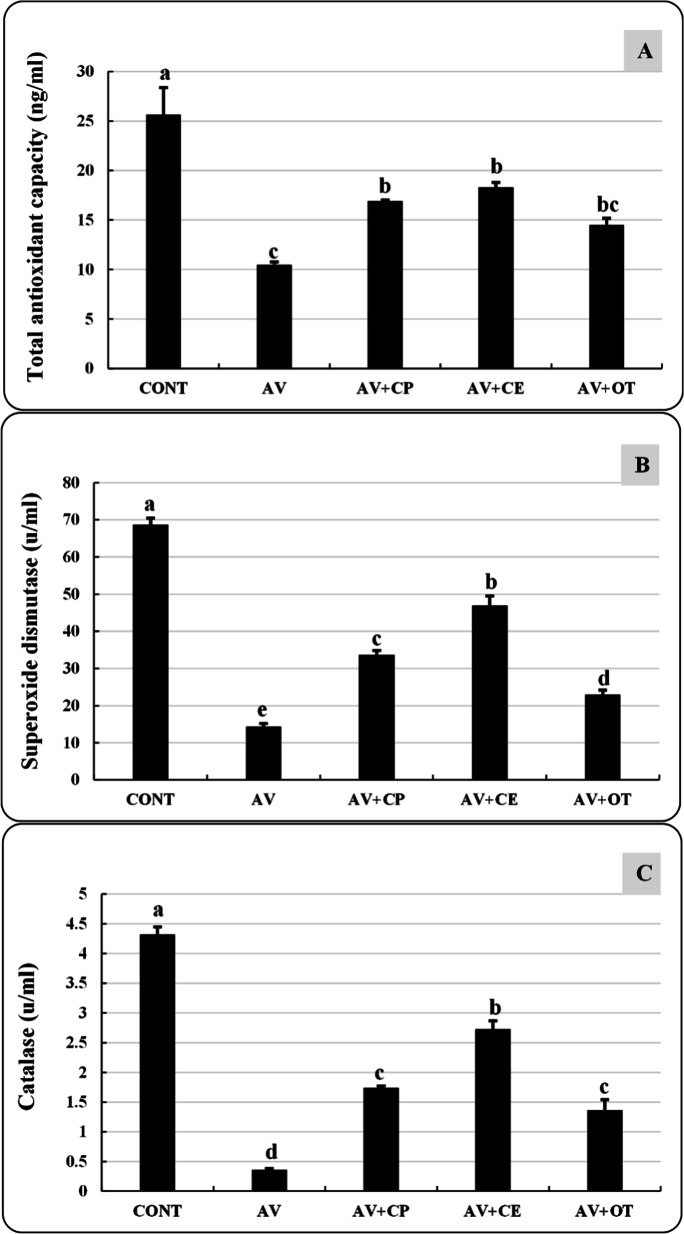
Total antioxidant capacity (ng/ml) (**A**), superoxide dismutase (u/ml) (**B**), and catalase (u/ml) (**C**) of *Aeromonas veronii*-infected *Oreochromis niloticus* treated for 15 days with *Coriandrum sativum* seed powder or its extract. The bars with different superscripts (a, b, c, d, and e) are significantly different (*P* < 0.05, one-way ANOVA). CONT (control group) fish not infected and fed with basal diet. AV: infected fish fed with basal diet. AV+CP: infected fish fed with diet supplemented with *Coriandrum sativum* seed powder (CP) at 30 mg/kg. AV+CE: infected fish fed with diet supplemented with *Coriandrum sativum* seed extract (CE) at 30 mg/kg. AV+OT: infected fish fed with diet supplemented with oxytetracycline (OT) at 500 mg/kg diet

TAC, SOD, and CAT levels were significantly lower in fish in the AV group than in the CONT group. Contrarily, these same parameters showed a considerable elevation in line with the patterns AV+CE> AV+CP and AV+OT but did not reach to the CONT group recorded values (Fig. 5).

### Immunological parameters

**Fig. 6:**
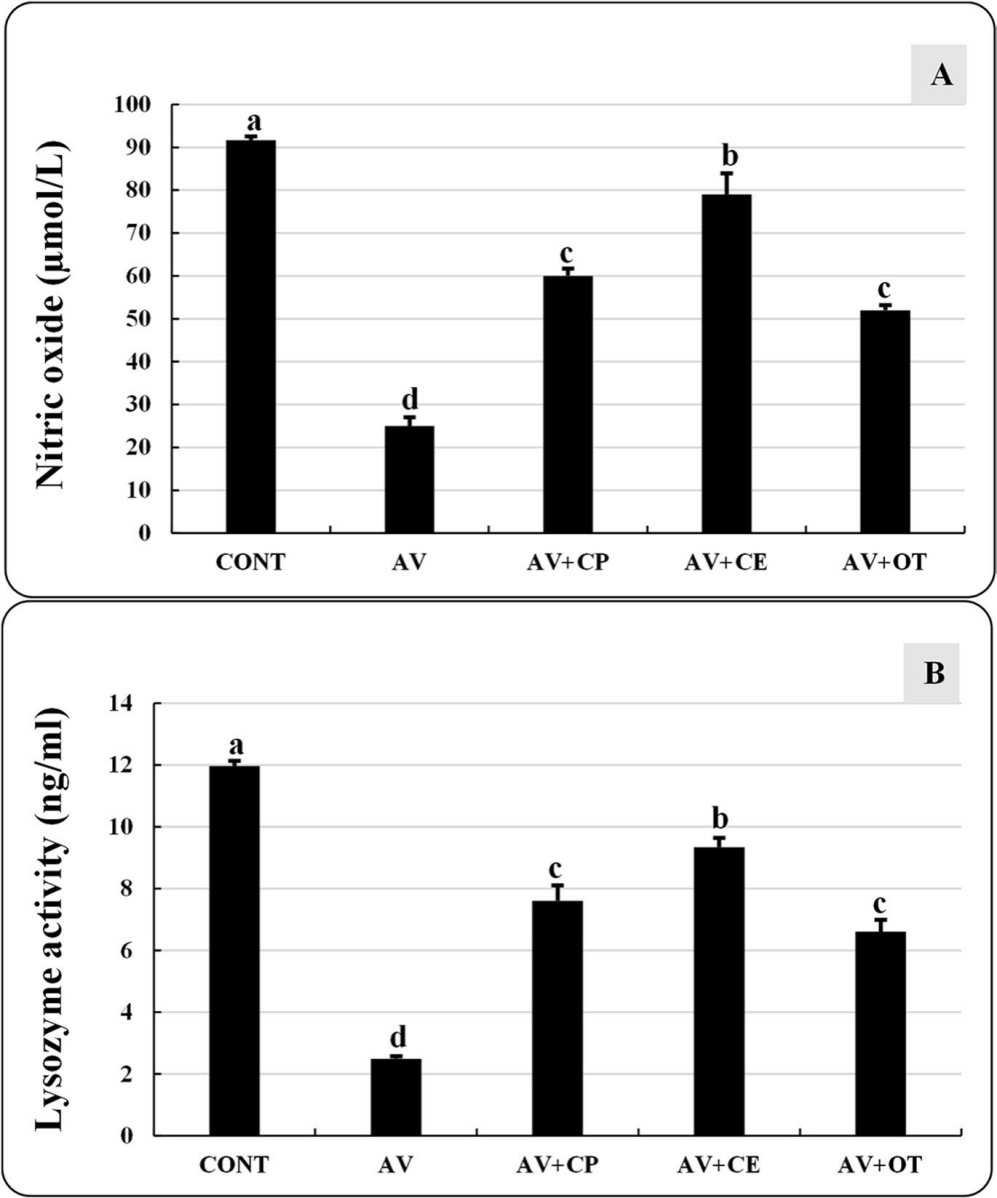
Nitric oxide (μmol/L) (**A**) and lysozyme activity (ng/ml) (**B**) of *Aeromonas veronii*-infected *Oreochromis niloticus* treated for 15 days with *Coriandrum sativum* seed powder or its extract. The bars with different superscripts (a, b, c, d, and e) are significantly different (*P* < 0.05, one-way ANOVA). CONT (control group) fish not infected and fed with basal diet. AV: infected fish fed with basal diet. AV+CP: infected fish fed with diet supplemented with *Coriandrum sativum* seed powder (CP) at 30 mg/kg. AV+CE: infected fish fed with diet supplemented with *Coriandrum sativum* seed extract (CE) at 30 mg/kg. AV+OT: infected fish fed with diet supplemented with oxytetracycline (OT) at 500 mg/kg diet

In comparison to the CONT group, the AV group showed a considerable drop in NO and LYZ activity. The NO and LYZ activities greatly improved in AV+CE, then AV+CP, and AV+OT, but they did not reach the levels seen in the CONT group. There were no significant differences seen in NO and LYZ activities between AV+CP and AV+OT groups (Fig. 6).

### Histopathological findings

**Fig. 7:**
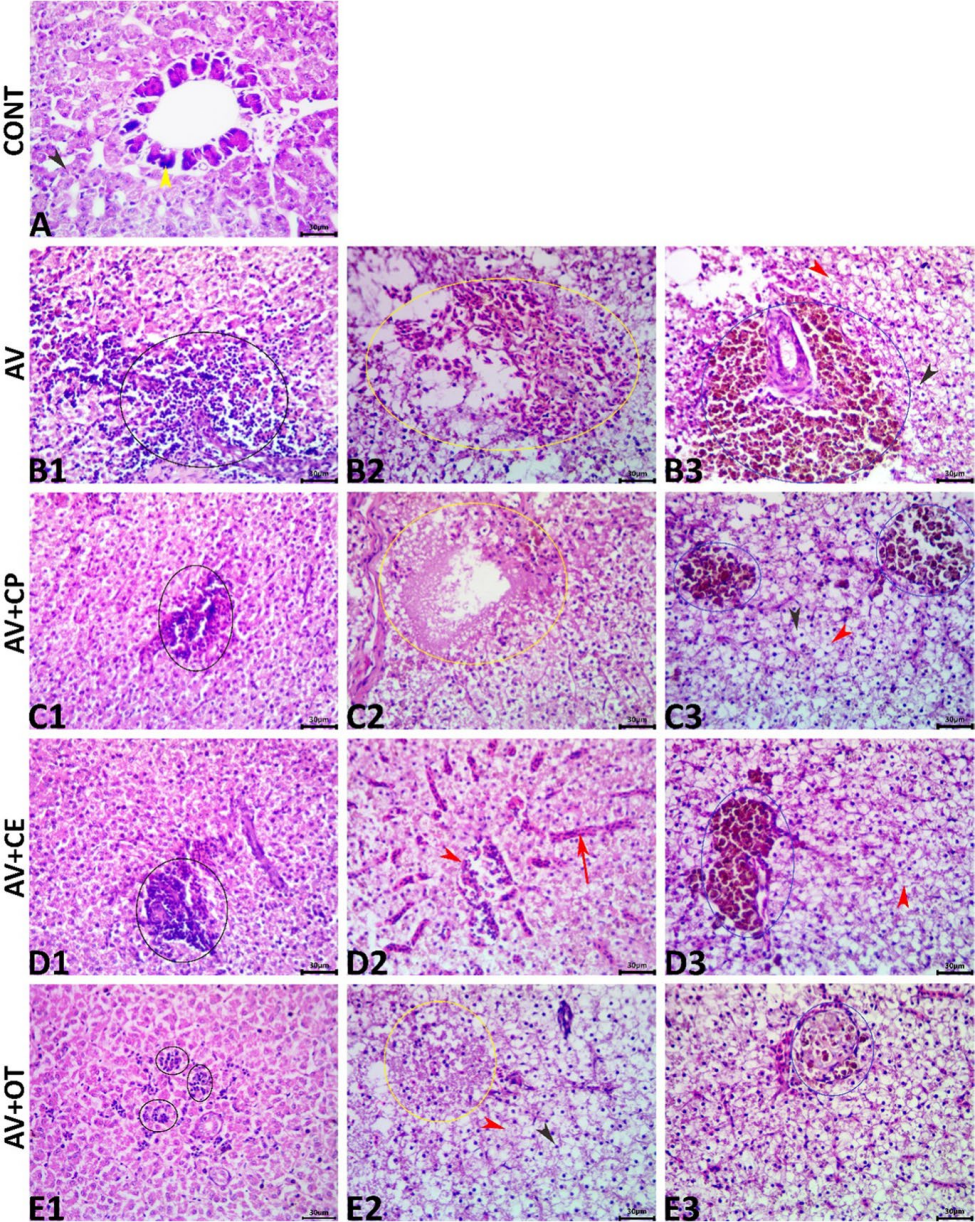
Representative photomicrograph of H&E-stained hepatic tissue sections showing a normal histological picture in the CONT fish (A); two-hepatocyte cords (black arrowhead) and exocrine pancreatic elements (yellow arrowhead). The AV group, infected fish shows notable inflammatory cell infiltrate (black ellipse) (B1), necrotic focus occupied by extravasated erythrocytes (yellow ellipse) (B2), and hyperplasia of melanomacrophage aggregates (blue ellipse), single-cell necrosis (red arrowhead), and (cytoplasmic lipoidal vacuolation (black arrowhead) (B3). The AV+CP group shows focal inflammatory cell aggregation (black ellipse) (C1), focal coagulative necrosis (yellow ellipse) (C2), and mild hyperplasia of the melanomacrophage aggregate (blue ellipses), single-cell necrosis (red arrowhead), and (cytoplasmic lipoidal vacuolation (black arrowhead) (C3). The AV+CE group shows focal inflammatory cell aggregation (black ellipse) (D1), congestion of the central vein (red arrowhead) and sinusoids (red arrow) (D2), and mild hyperplasia of the melanomacrophage aggregate (blue ellipse), and single-cell necrosis (red arrowhead) (D3). The AV+OT group shows minute inflammatory cell aggregations (black ellipses) (E1), focal coagulative necrotic focus (yellow ellipse), single-cell necrosis (red arrowhead), and (cytoplasmic lipoidal vacuolation (black arrowhead) (E2), and normal melanomacrophage aggregate (blue ellipse) (E3). scale bars 30 μm

The microscopic examination and image analysis of the livers of the control group declared normal hepatopancreatic architectures (Fig. 7A). Infection with *A. veronii* (AV group) induced a vast array of hepatopathic alterations mostly of inflammatory and circulatory nature with the absence of any neoplastic or preneoplastic changes. These alterations included but were not limited to notable inflammatory cell infiltrates, marked vascular and sinusoidal congestion, multifocal necrotic foci occasionally filled with extravasated blood, minute hemorrhages, intense lipoidal cytoplasmic vacuolations with cellular swelling and nuclear pyknosis, single-cell necrosis, and hyperplasia of the melanomacrophage aggregates (Figs. 7B1, B2, and B3). Supplementation with CP exerted weak hepatoprotective effects against the *A. veronii*-induced hepatopathy, as the same lesions recorded in the infected nontreated group were evident in the AV+CP-treated fish, but with lower severities (Figs. 7C1, C2, and C3). Supplementation with CE showed variable rescue effects against the *A. veronii*-induced hepatopathy, as a significant reduction in the inflammatory alterations, a moderate reduction in the circulatory and degenerative alterations, besides no effect on the hyperplasia of the melanomacrophage aggregates were observed in the AV+CE-treated fish compared to the infected nontreated group (Figs. 6D1, D2, and D3). Treatment with OT exhibited a sharp decline in the frequencies and severities of the inflammatory and circulatory alterations and the hyperplasia of the melanomacrophage aggregates, yet it somewhat increased the retrogressive alterations particularly the hepatocyte vacuolation and single-cell necrosis (Figs. 7E1, E2, and E3). The lesion frequencies, scores, and liver index of all groups were summarized in Table 5.

**Table 5:** Histopathological indices and lesion frequency of the liver *Aeromonas veronii*-infected *Oreochromis niloticus* treated for 15 days with *Coriandrum sativum* seed powder or its extract

Organ	Histopathological alteration			CONT		AV		AV+CP		AV+CE		AV+OT		*P*- value
	Reaction pattern	Type	w	FQ (%)	Index (w × a)	FQ (%)	Index	FQ (%)	Index	FQ (%)	Index	FQ (%)	Index	
Liver	Inflammatory alterations	- Inflammatory cell infiltrate	2	0	0.0 ± 0.0^b^	52	2.6 ± 0.7^a^	48	2.2 ± 0.6^a^	22	0.4 ± 0.2^b^	12	0.4 ± 0.2^b^	0.001
		- Granuloma formation	2	0	0.0 ± 0.0	4	0.2 ± 0.2	4	0.2 ± 0.2	0	0.0 ± 0.0	0	0.2 ± 0.2	0.736
	Circulatory alterations	-Vascular congestion	1	0	0.0 ± 0.0^b^	56	1.6 ± 0.5^a^	52	1.3 ± 0.4^a^	44	0.8 ± 0.3^ab^	30	1.7 ± 0.2^a^	0.018
		- Sinusoidal congestion	1	0	0.0 ± 0.0^b^	36	1 ± 0.4^a^	32	0.9 ± 0.3^ab^	22	0.4 ± 0.2^ab^	20	0.9 ± 0.2^ab^	0.118
		- Hemorrhages	2	0	0.0 ± 0.0	8	0.4 ± 0.4	8	0.4 ± 0.4	2	0.2 ± 0.2	8	0.2 ± 0.2	0.843
	Regressive alterations	- Acute cellular swelling	1	0	0.0 ± 0.0^b^	76	2.1 ± 0.7^a^	72	1.7 ± 0.7^ab^	42	0.5 ± 0.2^bc^	50	0.8 ± 0.2^abc^	0.021
		- Lipoidal cytoplasmic vacuolations	1	0	0.0 ± 0.0^b^	82	2.1 ± 0.5^a^	80	2.1 ± 0.5^a^	62	1.3 ± 0.4^a^	80	0.9 ± 0.2^ab^	0.002
		- Vacuolation foci	2	0	0.0 ± 0.0	6	0.2 ± 0.2	6	0.2 ± 0.2	4	0.2 ± 0.2	0	0.4 ± 0.2	0.719
		- Hepatocyte single-cell necrosis	2	0	0.0 ± 0.0^b^	36	1.6 ± 0.7^a^	32	1 ± 0.4^ab^	18	0.4 ± 0.2^b^	44	0.0 ± 0.0^b^	0.026
		- Hepatic necrotic foci	3	0	0.0 ± 0.0	12	0.6 ± 0.6	10	0.3 ± 0.3	6	0.3 ± 0.3	10	0.6 ± 0.4	0.772
		- Necrosis of the melanomacrophage aggregates	3	0	0.0 ± 0.0	0	0.0 ± 0.0	0	0.0 ± 0.0	0	0.0 ± 0.0	0	0.0 ± 0.0	-
	Progressive alterations	- Regenerated hepatocytes	2	0	0.0 ± 0.0	6	0.2 ± 0.2	8	0.4 ± 0.4	12	0.4 ± 0.4	6	0.2 ± 0.2	0.843
		- Hyperplastic cholangiocytes	2	0	0.0 ± 0.0	0	0.0 ± 0.0	0	0.0 ± 0.0	0	0.0 ± 0.0	0	0.0 ± 0.0	-
		- Basophilic foci	1	0	0.0 ± 0.0	4	0.2 ± 0.2	6	0.2 ± 0.2	8	0.2 ± 0.2	4	0.4 ± 0.2	0.719
		- Hyperplasia of melanomacrophage aggregates	2	0	0.0 ± 0.0	30	1.2 ± 0.6	32	1.4 ± 0.7	36	0.8 ± 0.3	36	0.4 ± 0.2	0.252
	Neoplastic alterations	- Adenomas	3	0	0.0 ± 0.0	0	0.0 ± 0.0	0	0.0 ± 0.0	0	0.0 ± 0.0	0	0.0 ± 0.0	-
		- Carcinomas	3	0	0.0 ± 0.0	0	0.0 ± 0.0	0	0.0 ± 0.0	0	0.0 ± 0.0	0	0.0 ± 0.0	-
Liver index					0.0 ± 0.0^c^		14.0 ± 3.31^a^		12.30 ± 3.06^ab^		5.9 ± 2.29^bc^		7.1 ± 1.77^abc^	0.001

Values are represented as the mean ± SE. The means within the same row carrying different superscripts are significant at *P* < 0.05

CONT (control group): fish not infected and fed with normal diet

AV: infected fish fed with normal diet

AV+CP: infected fish fed with diet supplemented with *Coriandrum sativum* seed powder (CP) at 30 mg/kg

AV+CE: infected fish fed with diet supplemented with *Coriandrum sativum* seed extract (CE) at 30 mg/kg

AV+OT: infected fish fed with diet supplemented with oxytetracycline (OT) at 500 mg/kg diet

*w* important factor, *a* score value, *FQ* frequency

**Fig. 8:**
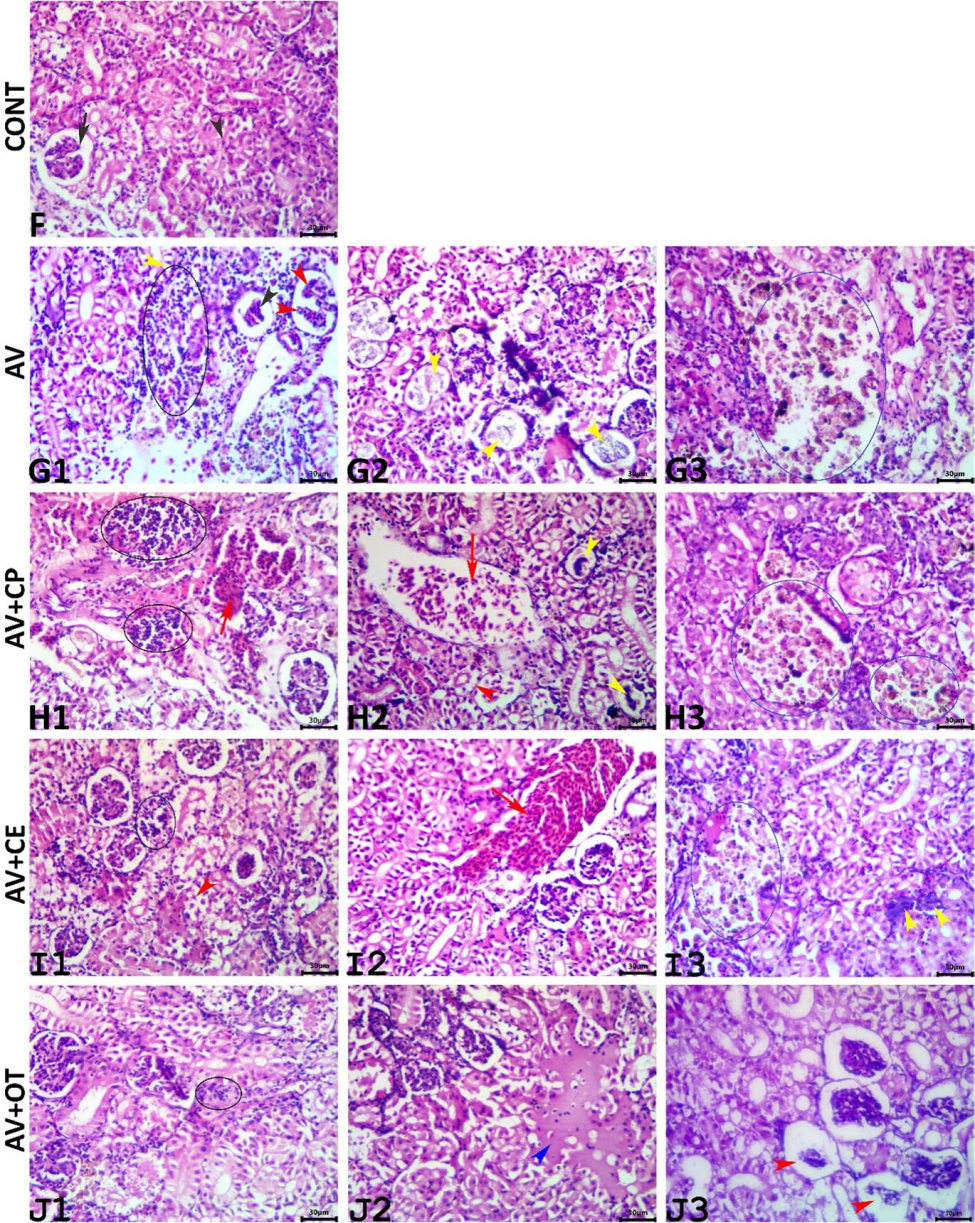
Representative photomicrograph of H&E-stained renal tissue sections show a normal glomerulus (black arrow) and renal tubule (black arrowhead) in the CONT fish (F). The AV group, the infected fish show inflammatory cell infiltrate (black ellipse), glomerular lobulation (red arrowheads), glomerular necrosis (black arrowhead), the vacuolated renal epithelium (yellow arrowhead) (G1), necrotic glomeruli (yellow arrowheads) (G2), and hyperplasia of melanomacrophage aggregates (blue ellipse) (G3). The AV+CP group shows inflammatory cell aggregates (black ellipses), vascular congestion (red arrow) (H1), necrotic glomeruli (yellow arrowheads), vascular congestion (red arrow), the necrotic tubular epithelium (red arrowhead) (H2), and hyperplasia of the melanomacrophage aggregate (blue ellipses) (H3). The AV+CE group shows minute inflammatory cell aggregation (black ellipse), detached tubular epithelium (red arrow) (I1), vascular congestion (red arrow) (I2), and hyperplasia of the melanomacrophage aggregate (blue ellipse) (I3). The AV+OT group shows minute inflammatory cell aggregation (black ellipse) (J1), interstitial edema (blue arrowhead) (J2), and necrotic glomeruli (red arrowheads) (J3). Scale bars 30 μm

The microscopic examination and image analysis of the kidneys of the control group declared normal renal architectures (Fig. 8F). Infection with *A. veronii* induced numerous nephropathic morphological alterations. Most of these alterations are less severe, but few tissue sections exhibited notable degenerative changes, collectively, these alterations included glomerulopathy (atrophy, lobulation, and necrosis), tubulopathy (vacuolation, necrosis, and cast formation), and the interstitial tissue lesions (edema, congestion, inflammatory cell infiltration, and hyperplasia of the melanomacrophage aggregates) (Figs. 8G1, G2, and G3). Supplementation with CP exerted non-significant rescue effects on the renal histology as most of the *A. veronii*-induced renal lesions were evident in the AV+CP-treated fish, with nearly the same reactive nature (Figs. 8H1, H2, and H3). Interestingly, supplementation with CE remarkably rescue the renal parenchyma in the AV+CE-treated fish against the *A. veronii*-induced nephropathic alterations, and many tubules showed regenerative reactions, yet the kidneys did not regain their normal histology. Numerous but mild lesions were still evident including atrophy of the glomerular tufts, vacuolation, detachment, and necrosis of the tubular epithelium, congestion of the interstitial vasculatures, and hyperplasia of the melanomacrophage aggregates (Figs. 8I1, I2, and I3). Treatment with OT possessed pros and cons effect, as it notably reduced both the severities and frequencies of the inflammatory, progressive, and most of the circulatory alterations caused by *A. veronii* infection, but it exaggerated the nephropathic degenerative alterations (Figs. 8J1, J2, and J3). The lesion frequencies, scores, and kidney index of all groups were summarized in Table 6.

**Table 6:** Histopathological indices and lesion frequency of the kidney *Aeromonas veronii*-infected *Oreochromis niloticus* treated for 15 days with *Coriandrum sativum* seed powder or its extract

Organ	Histopathological alteration			CONT		AV		AV+CP		AV+CE		AV+OT		*P*-value
	Reaction pattern	Type	w	FQ (%)	Index (w × a)	FQ (%)	Index	FQ (%)	Index	FQ (%)	Index	FQ (%)	Index	
Kidney	Inflammatory alterations	- Inflammatory cell infiltrate	2	0	0.0 ± 0.0	22	0.6 ± 0.4	22	0.6 ± 0.4	12	0.2 ± 0.2	6	0.2 ± 0.2	0.525
		- Granuloma formation	2	0	0.0 ± 0.0	0	0.0 ± 0.0	0	0.0 ± 0.0	0	0.0 ± 0.0	0	0.0 ± 0.0	-
	Circulatory alterations	- Interstitial congestion	1	0	0.0 ± 0.0	42	0.8 ± 0.4	40	0.8 ± 0.4	18	0.3 ± 0.2	12	0.2 ± 0.2	0.210
		- Interstitial edema	1	0	0.0 ± 0.0	8	0.2 ± 0.2	8	0.2 ± 0.2	4	0.2 ± 0.2	6	0.2 ± 0.2	0.908
		- Interstitial hemorrhage	2	0	0.0 ± 0.0	2	0.2 ± 0.2	2	0.2 ± 0.2	0	0.0 ± 0.0	2	0.2 ± 0.2	0.736
	Regressive alterations	- Collapsed glomeruli	2	0	0.0 ± 0.0	16	0.4 ± 0.2	14	0.4 ± 0.2	8	0.2 ± 0.1	10	0.2 ± 0.1	0.539
		- Necrotic glomeruli	3	0	0.0 ± 0.0	8	0.3 ± 0.3	8	0.3 ± 0.3	6	0.2 ± 0.2	10	0.1 ± 0.1	0.829
		- Vacuolation of the tubular epithelium	1	0	0.0 ± 0.0^b^	48	0.9 ± 0.3^a^	42	0.6 ± 0.2^ab^	24	0.2 ± 0.2^ab^	50	0.7 ± 0.2^ab^	0.075
		- Necrosis of the tubular epithelium	3	0	0.0 ± 0.0	18	0.6 ± 0.4	14	0.3 ± 0.3	6	0.3 ± 0.3	8	0.3 ± 0.3	0.719
		- Detached tubular epithelium	2	0	0.0 ± 0.0	20	0.4 ± 0.2	18	0.4 ± 0.2	12	0.2 ± 0.2	16	0.4 ± 0.2	0.643
		- Tubular dilatation	2	0	0.0 ± 0.0	0	0.0 ± 0.0	0	0.0 ± 0.0	0	0.0 ± 0.0	6	0.2 ± 0.2	0.418
		- Tubular cast formation	1	0	0.0 ± 0.0	6	0.2 ± 0.2	6	0.2 ± 0.2	2	0.0 ± 0.0	6	0.2 ± 0.2	0.736
		- Necrosis of the melanomacrophage aggregates	3	0	0.0 ± 0.0	0	0.0 ± 0.0	0	0.0 ± 0.0	0	0.0 ± 0.0	0	0.0 ± 0.0	-
	Progressive alterations	- Regenerated tubular epithelium	2	0	0.0 ± 0.0	12	0.2 ± 0.2	16	0.4 ± 0.2	18	0.6 ± 0.4	14	0.2 ± 0.2	0.544
		- Hyperplasia of the melanomacrophage aggregate	2	0	0.0±0.0	14	0.2 ± 0.2	14	0.2 ± 0.2	16	0.4 ± 0.2	22	0.4 ± 0.2	0.644
	Neoplastic alterations	- Adenomas	3	0	0.0 ± 0.0	0	0.0 ± 0.0	0	0.0 ± 0.0	0	0.0 ± 0.0	0	0.0 ± 0.0	-
		- Carcinomas	3	0	0.0 ± 0.0	0	0.0 ± 0.0	0	0.0 ± 0.0	0	0.0 ± 0.0	0	0.0 ± 0.0	-
Kidney index					0.0 ± 0.0		5.0 ± 1.63		4.6 ± 1.36		2.8 ± 1.73		3.5 ± 2.48	0.240

Values are represented as the mean ± SE. The means within the same row carrying different superscripts are significant at *P* < 0.05

CONT (control group): fish not infected and fed with normal diet

AV: infected fish fed with normal diet

AV+CP: infected fish fed with diet supplemented with *Coriandrum sativum* seed powder (CP) at 30 mg/kg

AV+CE: infected fish fed with diet supplemented with *Coriandrum sativum* seed extract (CE) at 30 mg/kg

AV+OT infected fish fed with diet supplemented with oxytetracycline (OT) at 500 mg/kg diet

*w* important factor, *a* score value, *FQ* frequency

## Discussion

Coriander leaves and seeds are rich sources of bioactive constituents (Laribi et al. 2015). The GC-MS analysis in this study showed that the methanolic extract of *C. sativum* included a large amount of L-linalool (92.52%), cis-linalool oxide (1.73%), trans-linalool oxide (1.7%), and other bioactive compounds, which indicated that the *C. sativum* seeds have bioactive compounds. Similar results were recorded in the CE, although the seeds were obtained from different localities. For example, seed extract from Kanpur contains 83.21% linalool (Bankar et al. 2011), seed extract from Tunisia contains 87.54% linalool (Msaada et al. 2007), and seed extract from Bangladesh contains 37.7% linalool (Bhuiyan et al. 2009). The differences in the extract of bioactive compounds can be related to the plant maturity stage (Msaada et al. 2007) as well as seasonal fluctuations, soil conditions, and environmental factors (Heywood 2002).

The CE demonstrated a large inhibition zone in this study against *A. veronii*, which indicated that the extract had a strong antibacterial effect. CE has bactericidal and antibacterial effects against several pathogens such as *A. veronii*, *A. hydrophila*, *Staphylococcus caseolyticus*, *S. aureus*, *Chromobacterium violaceum*, *Klebsiella pneumoniae*, *Pasteurella multocida*, *Escherichia coli*, and *Campylobacter* spp. (Abbas et al. 2022; Das et al. 2023; Duarte et al. 2016). The antibacterial effect of CE might be related to linalool and other bioactive components, which can damage the bacterial cell membrane and cause cell death as mentioned by Silva et al. (2011).

In this study, the clinical and postmortem results of the AV fish group were analyzed. These findings showed an increase in mucus secretion, eye turbidity, hemorrhagic patches with abnormalities in their swimming behavior, and congestion in hemopoietic organs (including the liver, spleen, and kidneys). These results are similar to those reported by Reda et al. (2022), Youssuf et al. (2020), Abd El Latif et al. (2019), and Raj et al. (2019) for *O. niloticus*, Cai et al. (2012) for the Chinese long-snout catfish, and Zhu et al. (2016) for the loach *Misgurnus anguillicaudatus.* The pathogenic symptoms and findings could be returned to cytotoxic and hemolytic activity and several exoenzymes (such as lipase, amylase, DNase, gelatinase, lecithinase, caseinase, and chitinase) produced by *A. veronii* (Sreedharan et al. 2011). In contrast, administering 30 mg/kg CE to the fish diet improved their health status and reduced *A. veronii* symptoms in the fish of the AV+CE group. This was the first study to show the protective effects of *C. sativum* against *A. veronii* in *O. niloticus*. Several studies have shown the prophylactic efficacy of CE against infection with numerous pathogenic bacteria in fish, such as *A. hydrophila* in *O. niloticus* and *Catla catla* (Das et al. 2023; Innocent et al. 2011) and *Yersinia ruckeri* in *Oncorhynchus mykiss* (Farsani et al. 2019). The results of the antibacterial activity showed that CE could significantly decrease the mortality rate and improve the general health conditions of the fish in the AV+CE group. In this study, *A. veronii* adversely affected the hematological parameters in the AV group, where significant declines in RBCs, HCT, Hgb, total protein, albumin, and globulin as well as leukopenia, neutropenia, and lymphopenia were observed. The decline in hematological parameters might be related to congestion and hemorrhage in hematopoietic organs, induced by *A. veronii* via the production of the hemolysin enzyme (Chandrarathna et al. 2018; Liu et al. 2022). Tort (2011) found that leukopenia and a considerable decrease in the differential leukocyte count are caused by prolonged and/or high stress (e.g., stress caused by *A. veronii* infection). Similar results were recorded in *O. niloticus* and *Cyprinus carpio* infected with *A. hydrophila* (Harikrishnan et al. 2003; Mukherjee et al. 2022). The current results showed considerable improvement in the hematological parameters of the infected group that was administered a diet supplemented with CE (AV+CE), followed by the groups that were administered AV+CP and AV+OT. This improvement might be due to the high levels of linalool present, which have antioxidant, anti-inflammatory, and antibacterial properties that mitigate the effects of *A. veronii*-induced hematologic alterations and promote the restoration of the functions of hematopoietic organs (Das et al. 2023; Laribi et al. 2015). Previous studies that performed coriander supplementation in fish diets as a prophylactic measure either found an increase in hematological markers or no change compared to the corresponding levels of the markers in the control groups. Das et al. (2023) recorded a significant increase in Hgb, MCV, MCH, and the thrombocyte count in *O. niloticus* that were administered a diet enriched with 2% coriander oil for 60 days, while no change occurred in the levels of WBC, RBC, HCT, and MCHC compared to their respective values in the control group. Innocent et al. (2011) found a substantial increase in the Hb content and serum protein level of *Catla catla* that were administered a diet supplemented with 2 g/kg CP for 14 days.

In the current results, the fish samples from the AV group exhibited a substantial increase in ALT, AST, ALP, urea, and creatinine compared to the control group. These results match those recorded by Reda et al. (2022) and Moustafa et al. (2020), who found that *O. niloticus* challenged by *A. veronii* and *A. hydrophila*, respectively, exhibited a significant increase in AST and ALT levels. In contrast, the current study found that the biochemical markers of the fish in the AV+CE group were significantly restored compared to the levels of the biochemical markers in the fish from the control, non-challenged group (CONT). Several studies have shown that an ethanolic extract of coriander enriched in flavonoids (isoquercetin and quercetin), phenolic compounds, and alkaloids has hepatic and renal protective effects (Laribi et al. 2015; Momin et al. 2012; Pandey et al. 2011).

The formation of reactive oxygen species (ROS) in response to microbial invaders is one of the first responses of the innate immune system of the host, which can stop bacteria from colonizing tissues (Spooner and Yilmaz 2011). Oxidative stress occurs when ROS production exceeds the antioxidant capacity, resulting in a cascade of biochemical and physiological alterations (Vani et al. 2021). Fish with septicemic diseases caused by *A. veronii* exhibit immunological suppression and oxidative damage due to very high levels of ROS-generating cytotoxicity (Krzymińska et al. 2011; Liu et al. 2022; Yang et al. 2020; Zhu et al. 2022). This clarified the findings of this study and the drop in the levels of TAC, SOD, and CAT enzymes in the AV group. Kurhalyuk and Tkachenko (2011) found that infections caused by *A. hydrophila* in sea trout (*Salmo trutta* L.) increased oxidative stress and compromised the hepatic antioxidant defense system. Baldissera et al. (2018) reported an alteration in the hepatic and renal antioxidant/oxidant balance in silver catfish following experimental infection with *A. caviae*. The most effective protection against oxidative stress-related cell damage is provided by antioxidant enzymes, which are catalyze the transformation of ROS and their byproducts into stable harmless compounds (Sáez and Están-Capell 2017). Antioxidant enzymes including SOD, glutathione peroxidase (GPx), and CAT are used by the innate defense mechanisms to combat ROS (Tang et al. 2013). Several studies have reported the antioxidant properties of CE, including its capacity to suppress ROS and absorb and neutralize free radicals (Wangensteen et al. 2004). Tannins, phenols, and flavonoids in CE have antiradical properties and can transform free radicals into more stable molecules through varies of mechanisms, for example, by acting as hydrogen and electron donors (Msaada et al. 2017). Therefore, the fish in the AV+CE group showed a greater improvement in the activities of the antioxidant enzymes, followed by those in the AV+CP and AV+OT groups.

The current findings showed in *O. niloticus* that were administered a diet supplemented with CE (AV+CE), the serum had higher levels of NO and lysozyme activity. This result might be related to the presence of linalool and other phenolic compounds in the CE, which was previously shown to play a key role in stimulating the fish immune system and the production of bioactive molecules (Abbas et al. 2022; Ahmed et al. 2020; Chang and Shen 2014; Das et al. 2023). Some studies that used CE as a prophylactic measure in aquafeeds also reported its ability to increase the immune capacity of fish to fight pathogenic bacteria in aquaculture. These studies reported an increase in the immune resistance of *O. niloticus* and *Catla catla* against *A. hydrophila* infection (Das et al. 2023; Innocent et al. 2011) and the resistance of *O. mykiss* against *Y. ruckeri* infection (Farsani et al. 2019).

In this study, the results of the histopathological analysis showed that the fish in the AV group had greater frequency of lesions and higher liver and kidney indices than those in the other infected groups that were administered therapy. Similar histopathological lesions were observed in the liver and kidneys of several fish species infected with *A. veronii*, including the Nile tilapia (Dong et al. 2017), crucian carp (Chen et al. 2019), largemouth bass (Pei et al. 2021), and channel catfish (Qin et al. 2022). In contrast, histopathological lesions in the liver and kidneys improved in the other infected groups that were administered therapy in the following order: AV+CE, followed by AV+OT and AV+CP. The improvement in the liver and kidney tissues of fish treated with CE might be related to the action of linalool, which has been shown in other studies to be a preventive and therapeutic agent against infection and chemical and drug-induced liver and kidney damage via its antioxidant and antibacterial effects (An et al. 2021; Bandeira Junior et al. 2021; Duarte et al. 2016; Mazani et al. 2022).

## Conclusion

The findings of this study indicated that *A. veronii* is one of the most harmful infections that seriously threatens the aquaculture industry, as it interferes with liver and kidney functions and weakens the antioxidant and immunological defense of *O. niloticus*. Administering 30 mg/kg coriander extract was effective in treating *A. veronii* infection in *O. niloticus*. These findings provided a new method for treating aquaculture diseases using a safe plant product that is readily available, eco-friendly, affordable, and not hazardous to human health. However, further studies need to be conducted to determine the extent to which coriander extract can be used for treating different life stages of fish and diseases in hatcheries in the presence of various stress factors, particularly overstocking in the ponds and climatic change.

## Data Availability

All data generated or analyzed during this study are included in this published article.

## References

[CR1] Abbas A (2022). Characterization of bioactives and nutra-pharmaceutical potential of supercritical fluid and hydro-distilled extracted coriander leaves essential Oil. Dose-Response.

[CR2] Abd El Latif AM, Elabd H, Amin A, Eldeen AIN, Shaheen AA (2019). High mortalities caused by *Aeromonas veronii*: identification, pathogenicity, and histopathologicalstudies in *Oreochromis niloticus*. Aquac Int.

[CR3] Ahmed SA, Reda RM, ElHady M (2020). Immunomodulation by *Coriandrum sativum* seeds (coriander) and its ameliorative effect on lead-induced immunotoxicity in Nile tilapia (*Oreochromis niloticus* L.). Aquac Res.

[CR4] Altmann J (1974). Observational study of behavior: sampling methods. Behaviour.

[CR5] Amal MNA, Koh CB, Nurliyana M, Suhaiba M, Nor-Amalina Z, Santha S, Diyana-Nadhirah KP, Yusof MT, Ina-Salwany MY, Zamri-Saad M (2018). A case of natural co-infection of Tilapia Lake Virus and *Aeromonas veronii* in a Malaysian red hybrid tilapia (*Oreochromis niloticus*×*O. mossambicus*) farm experiencing high mortality. Aquaculture.

[CR6] An Q, Ren J-N, Li X, Fan G, Qu S-S, Song Y, Li Y, Pan S-Y (2021). Recent updates on bioactive properties of linalool. Food Funct.

[CR7] Baldissera MD, Souza CF, Parmeggiani B, Leipnitz G, Verdi CM, Santos RV, Stefani LM, Baldisserotto B (2018). The disturbance of antioxidant/oxidant balance in fish experimentally infected by *Aeromonas caviae*: relationship with disease pathophysiology. Microb Pathog.

[CR8] Bandeira Junior G, de Souza CF, da Silva HNP, Bianchini AE, Rodrigues P, da Costa ST, Heinzmann BM, Cargnelutti JF, Baldisserotto B (2021). Combined effect of florfenicol with linalool via bath in combating *Aeromonas hydrophila* infection in silver catfish (*Rhamdia quelen*). Aquaculture.

[CR9] Bankar R, Kumar A, Puri S, Ali I, Sharma A, Khan IA (2011). Chemical composition and antimicrobial activity of essential oil from seed of *Coriandrum sativum* L. Anal Chem Lett..

[CR10] Bernet D, Schmidt H, Meier W, Burkhardt-Holm P, Wahli T (1999). Histopathology in fish: proposal for a protocol to assess aquatic pollution. J Fish Dis.

[CR11] Bhuiyan MNI, Begum J, Sultana M (2009). Chemical composition of leaf and seed essential oil of *Coriandrum sativum* L. from Bangladesh. Bangladesh J Pharmacol.

[CR12] Boyd CE, Tucker CS (2014). Handbook for aquaculture water quality.

[CR13] Cai S-H, Wu Z-H, Jian J-C, Lu Y-S, Tang J-F (2012). Characterization of pathogenic *Aeromonas veronii* bv. veronii associated with ulcerative syndrome from Chinese longsnout catfish (*Leiocassis longirostris* Günther). Braz J Microbiol.

[CR14] Chandrarathna HPSU, Nikapitiya C, Dananjaya SHS, Wijerathne CUB, Wimalasena SHMP, Kwun HJ, Heo G-J, Lee J, De Zoysa M (2018). Outcome of co-infection with opportunistic and multidrug resistant *Aeromonas hydrophila* and *A. veronii* in zebrafish: identification, characterization, pathogenicity and immune responses. Fish Shellfish Immunol.

[CR15] Chang MY, Shen YL (2014). Linalool exhibits cytotoxic effects by activating antitumor immunity. Molecules (Basel, Switzerland).

[CR16] Chen F, Sun J, Han Z, Yang X, Xian JA, Lv A, Hu X, Shi H (2019). Isolation, identification and characteristics of *Aeromonas veronii* from diseased crucian carp (*Carassius auratus gibelio*). Front Microbiol.

[CR17] Chen TC, Ormond RFG, Mok HK (2001). Feeding and territorial behaviour in juveniles of three co-existing triggerfishes. J Fish Biol.

[CR18] Citarasu T (2010). Herbal biomedicines: a new opportunity for aquaculture industry. Aquac Int..

[CR19] Dakhlaoui S, Wannes WA, Sari H, Hmida MB, Frouja O, Limam H, Tammar S, Bachkouel S, Jemaa MB, Jallouli S, Hessini K, Msaada K (2022). Combined effect of essential oils from lavender (*Lavandula officinalis* L.) aerial parts and coriander (*Coriandrum sativum* L.) seeds on antioxidant, anti-diabetic, anti-cancer and anti-inflammatory activities. J. Essent. Oil-Bear. Plants.

[CR20] Das S, Pradhan C, Pillai D (2023). Dietary coriander (*Coriandrum sativum* L) oil improves antioxidant and anti-inflammatory activity, innate immune responses and resistance to *Aeromonas hydrophila i*n Nile tilapia (*Oreochromis niloticus*). Fish Shellfish Immunol.

[CR21] Dien LT, Ngo TPH, Nguyen TV, Kayansamruaj P, Salin KR, Mohan CV, Rodkhum C, Dong HT (2023). Non-antibiotic approaches to combat motile aeromonas infections in aquaculture: current state of knowledge and future perspectives. Rev Aquac.

[CR22] Dong HT, Techatanakitarnan C, Jindakittikul P, Thaiprayoon A, Taengphu S, Charoensapsri W, Khunrae P, Rattanarojpong T, Senapin S (2017). Aeromonas jandaei and *Aeromonas veronii* caused disease and mortality in Nile tilapia, *Oreochromis niloticus* (L.). J Fish Dis.

[CR23] Duarte A, Luís Â, Oleastro M, Domingues FC (2016). Antioxidant properties of coriander essential oil and linalool and their potential to control Campylobacter spp. Food Control.

[CR24] Ellis AE (1990). Lysozyme assays. Techniques Fish Immunol.

[CR25] Farsani MN, Hoseinifar SH, Rashidian G, Farsani HG, Ashouri G, Van Doan H (2019). Dietary effects of *Coriandrum sativum* extract on growth performance, physiological and innate immune responses and resistance of rainbow trout (*Oncorhynchus mykiss*) against *Yersinia ruckeri*. Fish Shellfish Immunol.

[CR26] Fetcho JR, Binder MD, Hirokawa N, Windhorst U (2009). Startle Response. Encyclopedia of Neuroscience.

[CR27] Francis-Floyd R (1991). Aeromonas infections.

[CR28] Harikrishnan R, Nisha Rani M, Balasundaram C (2003). Hematological and biochemical parameters in common carp, *Cyprinus carpio*, following herbal treatment for *Aeromonas hydrophila* infection. Aquaculture.

[CR29] Hashem NMA, El-Son MAM, Ateya AI, Saleh RM (2022). Impact of lactoferrin supplementation on oxidative stress, gene expression and immunity dysfunction induced by Aeromonas veronii in Nile tilapia (*Oreochromis niloticus*). Aquacu Res.

[CR30] Heywood V, Şener B (2002). The conservation of genetic chemical diversity in medicinal aromatic plants. biomolecular aspects of biodiversity and innovative utilization.

[CR31] Innocent BX, MSA F, Dhanalakshmi (2011) Studies on the immouostimulant activity of *Coriandrum sativum* and resistance to *Aeromonas hydrophila* in *Catla catla*. J Appl Pharm Sci 1(7):132–135

[CR32] Krzymińska S, Tańska A, Kaznowski A (2011). Aeromonas spp. induce apoptosis of epithelial cells through an oxidant-dependent activation of the mitochondrial pathway. J Med Microbiol.

[CR33] Kuebutornye FKA, Abarike ED (2020). The contribution of medicinal plants to tilapia aquaculture: a review. Aquac Int.

[CR34] Kurhalyuk N, Tkachenko H (2011). Induction of oxidative stress and antioxidant defenses in the livers of sea trout, Salmo trutta L., with ulcerative dermal necrosis. Fisheries Aquatic Life.

[CR35] Laribi B, Kouki K, M'Hamdi M, Bettaieb T (2015). Coriander (*Coriandrum sativum* L.) and its bioactive constituents. Fitoterapia.

[CR36] Little EE, Finger SE (1990). Swimming behavior as an indicator of sublethal toxicity in fish. Environ. Toxicol. Chem..

[CR37] Liu G, Li J, Jiang Z, Zhu X, Gao X, Jiang Q, Wang J, Wei W, Zhang X (2022). Pathogenicity of *Aeromonas veronii* causing mass mortalities of *Odontobutis potamophila* and its induced host immune response. Fish Shellfish Immunol.

[CR38] Mandal S, Mandal M (2015). Coriander (*Coriandrum sativum* L.) essential oil: chemistry and biological activity. Asian Pac J Trop Biomed.

[CR39] Mazani M, Rezagholizadeh L, Shamsi S, Mahdavifard S, Ojarudi M, Salimnejad R, Salimi A (2022). Protection of CCl_4_-induced hepatic and renal damage by linalool. Drug Chem Toxicol.

[CR40] Momin AH, Acharya SS, Gajjar AV (2012). Coriandrum sativum-review of advances in phytopharmacology. Int J Pharm Sci Res.

[CR41] Moustafa EM, Dawood MAO, Assar DH, Omar AA, Elbialy ZI, Farrag FA, Shukry M, Zayed MM (2020). Modulatory effects of fenugreek seeds powder on the histopathology, oxidative status, and immune related gene expression in Nile tilapia (*Oreochromis niloticus*) infected with *Aeromonas hydrophila*. Aquaculture.

[CR42] Msaada K, Hosni K, Taarit MB, Chahed T, Kchouk ME, Marzouk B (2007). Changes on essential oil composition of coriander (*Coriandrum sativum* L.) fruits during three stages of maturity. Food Chem.

[CR43] Msaada K, Jemia MB, Salem N, Bachrouch O, Sriti J, Tammar S, Bettaieb I, Jabri I, Kefi S, Limam F, Marzouk B (2017). Antioxidant activity of methanolic extracts from three coriander (*Coriandrum sativum* L.) fruit varieties. Arab J Chem.

[CR44] Mukherjee D, Ghosal I, Marik A, Sen P, Chakraborty SB (2022). Mitigating *Aeromonas hydrophila* infection in Nile tilapia through dietary *Basella alba* and *Withania somnifera* supplementation: a bioassay-guided fractionation approach. Aquac Res.

[CR45] Neo YY, Parie L, Bakker F, Snelderwaard P, Tudorache C, Schaaf M, Slabbekoorn H (2015). Behavioral changes in response to sound exposure and no spatial avoidance of noisy conditions in captive zebrafish. Front Behav Neurosci.

[CR46] NRC (2011). (National Research Council) Nutrient requirements of fish and shrimp.

[CR47] Oprean R, Oprean L, Tamas M, Sandulescu R, Roman L (2001). Essential oils analysis. II. Mass spectra identification of terpene and phenylpropane derivatives. J Pharm Biomed Anal.

[CR48] Owen J, Iggo B, Scandrett F, Stewart CJBJ (1954). The determination of creatinine in plasma or serum, and in urine; a critical examination. Biochem J.

[CR49] Pachanawan A, Phumkhachorn P, Rattanachaikunsopon P (2008). Potential of *Psidium guajava* supplemented fish diets in controlling *Aeromonas hydrophila* infection in tilapia (*Oreochromis niloticus*). J Biosci Bioeng.

[CR50] Pandey A, Bigoniya P, Raj V, Patel KK (2011). Pharmacological screening of *Coriandrum sativum* Linn. for hepatoprotective activity. J Pharm Bioallied Sci.

[CR51] Patton C, Crouch S (1977). Enzymatic colorimetric method to determine urea in serum. Anal Chem.

[CR52] Pei C, Song H, Zhu L, Qiao D, Yan Y, Li L, Zhao X, Zhang J, Jiang X, Kong X (2021). Identification of *Aeromonas veronii* isolated from largemouth bass *Micropterus salmoides* and histopathological analysis. Aquaculture.

[CR53] Pessoa RBG, de Oliveira WF, Correia M, Fontes A, Coelho L (2022). Aeromonas and human health disorders: clinical approaches. Front Microbiol.

[CR54] Preena PG, Swaminathan TR, Kumar VJR, Singh ISB (2020). Antimicrobial resistance in aquaculture: a crisis for concern. Biologia.

[CR55] Qin G, Xu J, Ai X, Yang Y (2022) Isolation, identification, and pathogenicity of *Aeromonas veronii*, the causal agent of hemorrhagic septicemia in channel catfish (*Ictalurus punctatus*) in China. Fishes 7(6):394. 10.3390/fishes7060394

[CR56] Raj NS, Swaminathan TR, Dharmaratnam A, Raja SA, Ramraj D, Lal KK (2019). Aeromonas veronii caused bilateral exophthalmia and mass mortality in cultured Nile tilapia, *Oreochromis niloticus* (L.) in India. Aquaculture.

[CR57] Rajaraman V, Nonnecke B, Franklin S, Hammell D, Horst R (1998). Effect of vitamins A and E on nitric oxide production by blood mononuclear leukocytes from neonatal calves fed milk replacer. J Dairy Sci.

[CR58] Reda R, El-Murr A, Abd Elhakim Y, El-Shahat W (2021). Relationship between the productivity losses of tilapia and *Aeromonas Veronii* infection. Zagazig Vet J.

[CR59] Reda RM, El-Murr A, Abd Elhakim Y, El-Shahat W (2022). Aeromonas veronii detection in Egyptian fish farms with summer tilapia mortality outbreaks and the role of formic acid in limiting its spread. Aquac Res.

[CR60] Reitman S, Frankel S (1957). A colorimetric method for the determination of serum glutamic oxalacetic and glutamic pyruvic transaminases. Am J Clin Pathol.

[CR61] Reverter M, Bontemps N, Lecchini D, Banaigs B, Sasal PJA (2014). Use of plant extracts in fish aquaculture as an alternative to chemotherapy: current status and future perspectives. Aquaculture.

[CR62] Romero J, Feijoó CG, Navarrete P, Carvalho E (2012). Antibiotics in aquaculture–use, abuse and alternatives. Health and environment in aquaculture.

[CR63] Sáez GT, Están-Capell N, Schwab M (2017). Antioxidant Enzymes. Encyclopedia of Cancer.

[CR64] Saharia PK, Hussain IA, Pokhrel H, Kalita B, Borah G, Yasmin R (2021). Prevalence of motile aeromonas septicaemia (MAS) in fish culture systems of the Central Brahmaputra Valley Zone of Assam, India. Aquac Res.

[CR65] Serwecińska L (2020) Antimicrobials and antibiotic-resistant bacteria: a risk to the environment and to public health. Water 12. 10.3390/w12123313

[CR66] Silva F, Ferreira S, Queiroz JA, Domingues FC (2011). Coriander (*Coriandrum sativum* L.) essential oil: its antibacterial activity and mode of action evaluated by flow cytometry. J Med Microbiol.

[CR67] Spooner R, Yilmaz O (2011). The role of reactive-oxygen-species in microbial persistence and inflammation. Int J Mol Sci.

[CR68] Sreedharan K, Philip R, Bright Singh IS (2011). Isolation and characterization of virulent *Aeromonas veronii* from ascitic fluid of oscar *Astronotus ocellatus* showing signs of infectious dropsy. Dis Aquat Org.

[CR69] Suresh T, Nithin MS, Kushala KB, Girisha SK, Shivakumar VB, Dheeraj SB, Puneeth TG, Kishan K, Vinay TN (2023). Largescale mortality of *Oreochromis mossambicus* in lakes and reservoirs of Karnataka due to coinfection of Tilapia Lake virus (TiLV) and multidrug-resistant *Aeromonas veronii*: an emerging fish disease in India. Aquaculture.

[CR70] Suvarna KS, Layton C, Bancroft JD (2018). Bancroft’s theory and practice of histological techniques E-Book.

[CR71] Tabuti JRS, Lye KA, Dhillion SS (2003). Traditional herbal drugs of Bulamogi, Uganda: plants, use and administration. J Ethnopharmacol.

[CR72] Tang ELH, Rajarajeswaran J, Fung SY, Kanthimathi MS (2013). Antioxidant activity of *Coriandrum sativum* and protection against DNA damage and cancer cell migration. BMC Complement Altern Med.

[CR73] Tenover FC, Schmidt TM (2019). Antimicrobial Susceptibility Testing☆. Encyclopedia of microbiology.

[CR74] Tort L (2011). Stress and immune modulation in fish. Dev Comp Immunol.

[CR75] Vani R, Carl H, Masannagari P, Kaneez Fatima S (2021). Modulations in oxidative stress of erythrocytes during bacterial and viral infections. Erythrocyte.

[CR76] Wang Y, Guo J-l, Li K, Bureau DP (2006). Effects of dietary protein and energy levels on growth, feed utilization and body composition of cuneate drum (Nibea miichthioides). Aquaculture.

[CR77] Wangensteen H, Samuelsen AB, Malterud KE (2004). Antioxidant activity in extracts from coriander. Food Chem.

[CR78] Wei D, Bao E, Wen Y, Zhu S, Ye Z, Zhao J (2021). Behavioral spatial-temporal characteristics-based appetite assessment for fish school in recirculating aquaculture systems. Aquaculture.

[CR79] White SL, Volkoff H, Devlin RH (2016). Regulation of feeding behavior and food intake by appetite-regulating peptides in wild-type and growth hormone-transgenic coho salmon. Horm Behav.

[CR80] Yang B, Song H, An D, Zhang D, Raza SH, Wang G, Shan X, Qian A, Kang Y, Wang C (2020) Functional analysis of preA in *Aeromonas veronii* TH0426 reveals a key role in the regulation of virulence and resistance to oxidative stress. Int J Mol Sci 21. 10.3390/ijms21010098 10.3390/ijms21010098 PMC6981600 31877791

[CR81] Youssuf H, Abdel Gawad E, El Asely AM, Elabd H, Matter A, Shaheen A, Abbass A (2020). Insight into summer mortality syndrome in farmed Nile tilapia (*Oreochromis niloticus*) associated with bacterial infection. Benha Med J.

[CR82] Zhu M, Wang XR, Li J, Li GY, Liu ZP, Mo ZL (2016). Identification and virulence properties of *Aeromonas veronii* bv. sobria isolates causing an ulcerative syndrome of loach *Misgurnus anguillicaudatus*. J Fish Dis.

[CR83] Zhu X, Qian Q, Wu C, Zhu Y, Gao X, Jiang Q, Wang J, Liu G, Zhang X (2022) Pathogenicity of *Aeromonas veronii* causing mass mortality of largemouth bass (*Micropterus salmoides*) and its induced host immune response. Microorganisms 10. 10.3390/microorganisms10112198 10.3390/microorganisms10112198 PMC9699015 36363790

